# FAR-RED INSENSITIVE 219/JAR1 Contributes to Shade Avoidance Responses of *Arabidopsis* Seedlings by Modulating Key Shade Signaling Components

**DOI:** 10.3389/fpls.2017.01901

**Published:** 2017-11-02

**Authors:** Swadhin Swain, Han-Wei Jiang, Hsu-Liang Hsieh

**Affiliations:** Institute of Plant Biology, College of Life Science, National Taiwan University, Taipei, Taiwan

**Keywords:** shade avoidance response, FIN219/JAR1, jasmonates, shade-induced hypocotyl elongation, *Arabidopsis*, shade signaling

## Abstract

To receive an ample amount of light, plants use elongation growth in response to vegetation shade. The combined interaction of light and hormones, including jasmonic acid (JA) signaling controls this elongation. However, the detailed molecular mechanisms underlying the response are still emerging. FAR-RED INSENSITIVE 219/JASMONATE RESISTANCE 1 (FIN219/JAR1), a cytoplasmic localized JA-conjugating enzyme, integrates far-red light and JA signaling. Here, we report that FIN219/JAR1 negatively regulates shade-induced hypocotyl elongation and gene expression in *Arabidopsis* seedlings in response to shade. In turn, simulated shade reduces FIN219 protein accumulation. Analysis of *phyA 211 fin219-2* double mutants indicated that FIN219 and phyA are synergistic in regulating shade-induced hypocotyl elongation and gene expression. Moreover, FIN219 differentially affected the expression of the shade-signaling bHLH factors PIF5 and PAR1, thereby increasing the expression of the auxin-response genes *IAA29* and *SAUR68* on exposure to shade. Furthermore, the protein level of CONSTITUTIVE PHOTOMORPHOGENIC 1 (COP1) was affected in both *fin219* mutants and overexpression lines as compared with the wild type under shade. Intriguingly, ectopic expression of *FIN219* inhibited the nuclear accumulation of COP1 in response to shade. Further co-immunoprecipitation studies revealed that FIN219 interacted with COP1 and phyA under shade. Therefore, FIN219/JAR1 may play a vital role in modulating the *Arabidopsis* response to simulated shade via multiple layers of molecular mechanisms.

## Introduction

Light carries most of the essential information needed for plant growth and development (Von Arnim and Deng, 1996; Fankhauser and Chory, 1997; Chory, 2010). Photoreceptors such as phytochromes, cryptochromes, and UVR8 perceive light as a developmental signal (Li et al., 2011; Liu et al., 2011; Tilbrook et al., 2013). Vegetation shade reduces plants’ access to sufficient light. Shade-intolerant plants such as *Arabidopsis* trigger a suite of responses, collectively called shade avoidance syndrome (SAS), including elongation of hypocotyls, stems and petioles, hyponasty (upward bending of leaves) and early flowering (Franklin, 2008; Casal, 2012). SAS is a default developmental program and suppressed under normal light by phyB along with phyD and phyE (Franklin and Quail, 2010; Casal, 2013).

Phytochromes exist in two forms: the red light (666 nm)-absorbing P_r_ form, and far-red light (730 nm)-absorbing P_fr_ form (Li et al., 2011). However, monochromatic red (R) or far-red (FR) light usually does not convert 100% of P_r_ to P_fr_ or vice versa. Thus, a dynamic equilibrium is established between the two phy forms (P_r_ and P_fr_) depending on the quality of light (Possart et al., 2014). Under white light (high R:FR), the active phyB, P_fr_, migrates to the nucleus and interacts with various PIFs and drives their degradation (Lorrain et al., 2008). The onset of low R:FR ratio shifts the steady-state equilibrium toward the inactive Pr form (Franklin and Quail, 2010). As a result, PIFs become stabilized and more abundant (Lorrain et al., 2008). Genome-wide analysis revealed that PIF4 and PIF5 preferentially bind to auxin biosynthetic and signaling gene promoters (Hornitschek et al., 2012; Leivar et al., 2012b) and activate their expression under low R:FR light. PIF7 also functions as a major regulator for shade-induced hypocotyl elongation and is dephosphorylated in response to shade (Li et al., 2012). HFR1 physically interacts with PIF4 and PIF5 and inhibits their binding to the target promoters, thus negatively regulating shade responses (Hornitschek et al., 2009).

CONSTITUTIVE PHOTOMORPHOGENIC 1 (COP1) is a repressor of photomorphogenesis and encodes an RING-finger E3 ubiquitin ligase. Under darkness, COP1 accumulates in the nucleus and targets degradation of positive regulators of photomorphogenesis such as HY5, HYH, LAF1, and HFR1 (Deng et al., 1991; Ballesteros et al., 2001; Lorrain et al., 2006). As well, COP1 is required for activating shade signaling. Mutants of *COP1* can barely sense vegetation shade, whereas overexpression of *COP1* leads to a constitutive shade avoidance phenotype (Pacín et al., 2013). The molecular mechanisms of COP1-mediated shade responses are emerging. COP1 rapidly accumulates in the nucleus under low R:FR, and this nuclear accumulation is essential for full shade responses (Pacín et al., 2013). Further evidence indicates that COP1 enhances HFR1 degradation under shade, leading to an increase of PIF-mediated gene expression, auxin levels, and thus stem growth (Pacín et al., 2016). COP1 and SPA1 together participate in hypocotyl and leaf petiole elongation in response to low R:FR by targeting the HFR1degradation by 26S proteasome (Rolauffs et al., 2012). The *cop1 bbx21 bbx22* triple mutant is responsive to the shade, which suggests that the shade insensitivity phenotype of *cop1* is mediated through both *BBX21* and *BBX22* (Crocco et al., 2010). However, how COP1 regulates SAS at the molecular level needs further investigation.

Phytohormones play important roles in light control of plant development (Lau and Deng, 2010; Kurepin and Pharis, 2014). The interplay of jasmonates (JAs) and light signaling is accepted (Kazan and Manners, 2011; Hsieh and Okamoto, 2014). Although low R:FR light perturbs JA signaling (Moreno et al., 2009; Robson et al., 2010; Cerrudo et al., 2012; De Wit et al., 2013; Chico et al., 2014; Leone et al., 2014), modulation of shade signaling by JA or its signaling components is still under examination. Recent evidence has revealed the interplay between JAs and shade, which is critical for growth-defense balance. This process may involve resource allocation between growth and immunity (Yang et al., 2012; Ballaré, 2014; Mazza and Ballaré, 2015).

The *jar1* mutants show reduced sensitivity to exogenous JA and enhanced susceptibility against soil fungus, *Pythium irregulare* (Staswick et al., 1992, 1998). Later, *jar1-1* was mapped to the same locus as *fin219-1* (Staswick et al., 2002), a suppressor of *cop1* mutant under darkness (Hsieh et al., 2000). FIN219 (GH3.11) belongs to a GH3 family of proteins, and its expression is rapidly induced by auxin (Hsieh et al., 2000). *FIN219/JAR1* encodes JA-amino-synthetase, which conjugates isoleucine (Ile) with jasmonic acid (JA) to form the bioactive jasmonoyl-L-isoleucine (JA-Ile) (Staswick and Tiryaki, 2004). JA-Ile is an oxylipin that profoundly affects plant developmental and stress responses (Bari and Jones, 2009; Browse, 2009).

The *fin219/jar1* mutant exhibits an insensitive long-hypocotyl phenotype under continuous far-red (cFR) light, which indicates its role in phyA-mediated signaling (Hsieh et al., 2000; Chen et al., 2007). Moreover, FIN219/JAR1 physically interacts with COP1 under darkness and cFR light and negatively regulates COP1 under FR light. Artificial induction of FIN219 protein accumulation via overexpression inhibited COP1 nuclear localization and hence stabilized HY5 protein (Wang et al., 2011). In addition, FIN219 might contribute to signaling of other hormones such as auxin, ethylene, gibberellin and abscisic acid by regulating a large number of bHLH transcription factors (TFs) (Chen et al., 2015).

Under low R:FR light, both *phyA* and *fin219* mutant seedlings show enhanced hypocotyl elongation as compared with the wild type (Johnson et al., 1994; Yanovsky et al., 1995; Robson et al., 2010). Although the *phyA* phenotype is attributed to a FR light-mediated high irradiance response, the molecular mechanism underlying *fin219*-mediated shade avoidance phenotype is still under debate.

In the present study, we examined the functional significance of *FIN219* under simulated shade in *Arabidopsis* seedlings. With a combination of genetic and biochemical experiments, we demonstrate that *FIN219* negatively regulates shade avoidance responses by modulating key shade signaling components. Moreover, *FIN219*-mediated shade avoidance responses were independent of phyA-mediated high irradiance responses.

## Materials and Methods

### Plant Materials and Growth Conditions

The *fin219-2* (SALK_059774), *fin219-1, jar 1-1, phyA 211, cop1-4, cop1-6, cop1-4 fin219-2*, and *cop1-6 fin219-2* mutants and glucocorticoid-inducible *FIN219* transgenic line (*pGR:FIN219*; *PGR219*) were described previously (Hsieh et al., 2000; Chen et al., 2007; Wang et al., 2011). *pGR219* seedlings were harvested in GM plates with 1 μM dexamethasone (Dex) to induce *FIN219* expression. The *phyA 211 fin219-2* double mutant was generated by crossing *phyA 211* with the *fin219-2* mutant and selecting homozygous plants in an F2 generation by T-DNA-specific primers. All mutants are in a Col-0 background. Seeds of *Arabidopsis thaliana* were surface-sterilized and plated on growth medium (1/2 strength Murashige and Skoog medium, Duchefa Biochemie; 0.3% sucrose, 0.5% MES, 0.5% agar). After 3 days of incubation at 4°C, seed plates were kept at 22°C, 150 μmol m^-2^s^-1^ fluorescence white light for 16 h for germination, then transferred to an LED growth chamber (HIPONT, 721FTEC, Taiwan). Germinating seedlings were grown in continuous white light (high R:FR) for 2 days, then kept in continuous white light or transferred to continuous simulated shade (low R:FR) for an additional 4 days.

### Light Measurements

The HIPONT, 721FTEC (Taiwan) growth chamber equipped with monochromatic LED light sources for white light (455 nm), red light (650 nm), and far-red light (730 nm) (Supplementary Figure S4), was used for shade avoidance study. In our study, white light and simulated shade represented high red:far-red light ratio (R5.21 μmol m^-2^s^-1^/FR2.25 μmol m^-2^s^-1^ ∼ 2.3) and low red:far-red ratio (R5.21 μmol m^-2^s^-1^/80.02 μmol m^-2^s^-1^ ∼ 0.06), respectively. Photon fluencies were estimated with use of a Li-Cor (LI-250A, LiCor Corp., Lincoln, NE, United States) and spectral distribution was measured by an Ocean Optics spectrum meter (USB2000, Florida, United States). Photosynthetically active radiation (white + red light) was kept constant at 70 μmol m^-2^s^-1^.

### Hypocotyl Length Measurements and Western Blot Quantification

Digital images were analyzed by the US National Institutes of Health ImageJ software (Bethesda, MD, United States^1^) to measure lengths of hypocotyl and to quantify Western blot band intensities.

### RNA Extraction, cDNA Synthesis, and Quantitative Real-Time PCR

RNA extraction and cDNA synthesis were as described previously (Chen et al., 2015). Samples of 100 mg were ground in 0.5 ml Buffer A (1MTris-HCl, pH 7.3, 5 mMEDTA, pH 8.0, 1% SDS), then extracted twice with an equal volume of phenol and once with chloroform:isoamylalcohol (24:1). The supernatant was precipitated with LiCl (final working concentration 3 M) and incubated at -20°C overnight. After centrifugation, the pellet was dissolved completely in 0.5 ml 2% potassium acetate, then precipitated again with isopropanol. Total RNA (2 μg) was treated with DNase to prevent genomic DNA contamination, then used as a template for cDNA synthesis with the ABI cDNA transcription kit (#4368814). Real-time PCR involved CFX96 Touch Real-time PCR Detection System (Bio-Rad, United States). Gene-specific primers (Supplemental Table S1) were used for analyzing mRNA levels of *ACT2* (AT3G18780), *PIL1* (AT2G46970), *ATHB2* (AT4G16780), *FIN219* (AT2G46370), *HFR1* (AT1G02340), *PAR1* (AT2G42870) and *PIF5* (AT3G59060), *IAA29* (AT4G32280) and *SAUR68* (AT1G29490) by qPCR. *ACT2* was used as internal control to normalize the expression levels, then standardized to the wild-type level under white light.

### Protein Extraction and Protein Gel Blot Analysis

Total protein was extracted with extraction buffer (50 mM Tris-HCl, pH 7.5, 150 mM NaCl, 10 mM MgCl_2_, 0.1% NP-40, 1 mM PMSF and 1X protease inhibitor) as described (Hsieh et al., 2000). Total protein, 150 μg, was loaded in each lane and separated on 8% SDS-PAGE and transferred to PVDF membrane (Millipore). Protein gel blot analyses involved standard methods (Sambrook and Russell, 2001) with FIN219, PHYA and α-tubulin monoclonal, and COP1 polyclonal antibodies.

### Protoplast Isolation and Subcellular Localization Study

*Arabidopsis* mesophyll protoplast isolation and transfection were performed as described (Yoo et al., 2007). In brief, 4-week-old well-expanded leaves were peeled (Wu et al., 2009) and incubated in enzyme solution (20 mM MES, 1.5% cellulase R10, 0.4% macerozyme R10, 0.4 M mannitol, 20 mM KCl, 10 mM CaCl2, 5 mM b-mercaptoethanol, and 0.1% bovine serum albumin, pH 5.7) for 1 h. Protoplasts were collected by centrifugation at 100 × *g* and washed twice with W5 solution (2 mM MES, 154 mM NaCl, 125 mM CaCl2, and 5 mM KCl, pH 5.7). Protoplasts were resuspended in W5 solution and incubated on ice for at least 30 min, then washed with MMG solution (4 mM MES, 0.4 M mannitol, and 15 mM MgCl_2_, pH 5.7) and resuspended at 2 × 10^5^ cells mL^-1^ in MMG solution. For protoplast transfection, 200 μL protoplasts was mixed with 10 μg (∼20 μL) DNA and 220 μL PEG solution (40% polyethylene glycol 4000, 0.2 M mannitol, and 100 mM CaCl2), then transfected protoplasts were washed twice, resuspended in W5 solution with 1 μM Dex and incubated overnight in the dark. On the next day, the protoplasts were treated with white light and shade light. The GFP fusion construct *p35S:GFP-COP1* was used for transfection. The nuclei of protoplasts were stained with 4′,6-diamidino-2-phenylindole. Transformed protoplasts were visualized by confocal laser scanning microscopy (Leica TCS SP5 Confocal Spectral Microscope) and image processing involved LAS AF lite software from Leica.

### Co-immunoprecipitation Analysis

Co-immunoprecipitation analysis was performed as described (Chen et al., 2007). The seedlings were ground with grinding buffer (50 mM Tris-HCl, pH 5.7, 150 mM NaCl, 10 mM MgCl_2_, 0.1% bovine serum albumin, 0.1% Nonidet P-40, 1 mM PMSF, 2X Protease Inhibitor Cocktail [Sigma], and 50 μM MG132). A total of 2 mg of proteins was mixed with beads and incubated at 4°C for 4 h, then washed three times with same grinding buffer. Pellets were analyzed by standard SDS-PAGE and subjected to protein gel blot analysis.

## Results

### *FIN219* Negatively Regulates Hypocotyl Elongation and Gene Expression under Simulated Shade

**FIGURE 1:**
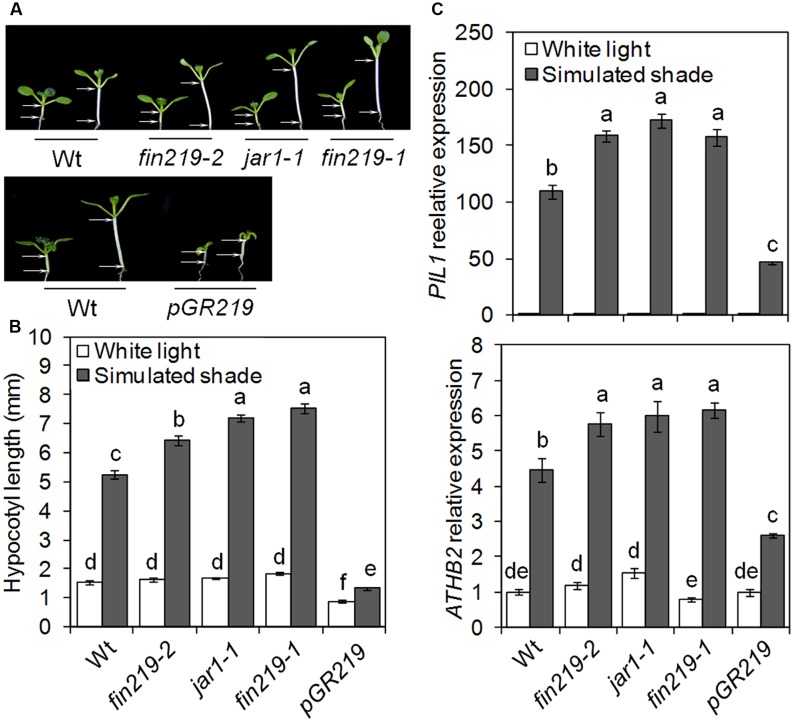
*FIN219* negatively regulates shade-induced hypocotyl elongation and gene expression. **(A)** Seedlings of *fin219* mutants (*fin219-2, jar1-1, fin219-1*) and *FIN219* overexpression line (*pGR219*) were grown under white light (high R:FR) or simulated shade (low R:FR) for 4 days and then photographs were taken. **(B)** Quantification of hypocotyl lengths of seedlings grown in **A**. Data are mean ± SE (*n* = 25). **(C)** Quantitative RT-PCR (qRT-PCR) analyses of *PIL1* and *ATHB2* expression in seedlings grown under white light or simulated shade for 4 days standardized to the wild type under white light. Data are mean ± SE from three biological replicates. Different lowercase letters represent significant differences by ANOVA at *P* < 0.05.

Jasmonic acid biosynthetic and signaling mutants display enhanced shade-induced hypocotyl elongation (Robson et al., 2010). We previously demonstrated that FIN219/JAR1 integrates phyA-mediated FR light and JA signaling (Wang et al., 2011; Chen et al., 2015). To further understand the role of FIN219 in the shade avoidance response, we examined hypocotyl elongation and gene expression responses under white light (high R:FR) and simulated shade (low R:FR). Seven-day-old seedlings of *fin219* mutants [*fin219-2* (Wang et al., 2011), *jar1-1* (Staswick et al., 1998), and *fin219-1* (Hsieh et al., 2000)], and overexpression of *FIN219* in a *fin219-2* genetic background driven by glucocorticoid-inducible promoter [*pGR:FIN219*, also pGR219 (Wang et al., 2011)] were compared with the corresponding wild type. Under white light, *fin219* mutants showed similar hypocotyl length to that of the wild type. However, under simulated shade, the hypocotyl was longer for all *fin219* mutants than the wild type [129.7% (*fin219-2*), 180.2% (*jar1-1*) and 197.6% (*fin219*-1), *p* < 0.01] (**Figures 1A,B**). The *FIN29* overexpression line, *pGR219*, induced by Dex had a short-hypocotyl phenotype under white light. Although *pGR219* responded to low R:FR light, hypocotyl elongation was lower than that of the wild type (**Figures 1A,B**). Furthermore, we quantified the expression of shade-inducible marker genes *PIL1* and *ATHB2.* Under shade, the expression of these genes was induced in the wild type. Under white light, their expression in the *fin219* mutants and *pGR219* was largely similar to that of the wild type. However, under simulated shade, the expression was enhanced in the *fin219* mutants but significantly reduced in *pGR219* (**Figures 1C,D**). FIN219 may inhibit shade-induced hypocotyl elongation and gene expression.

### Simulated Shade Reduces FIN219 Protein Level and FIN219 Affects phyA and COP1 Protein Accumulation

**FIGURE 2:**
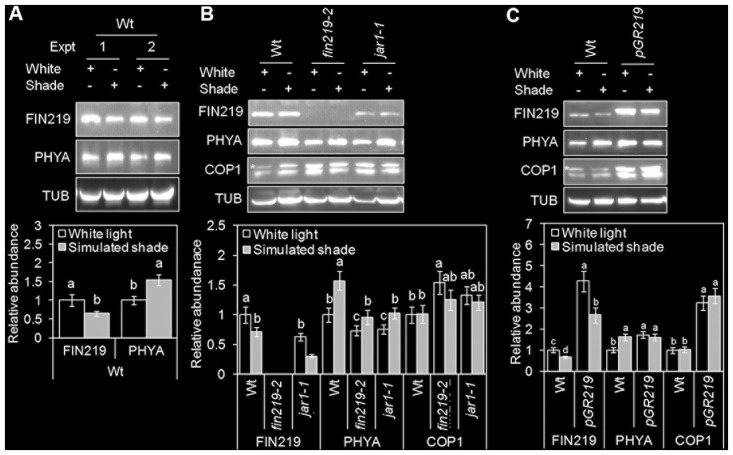
FIN219 levels reduced under simulated shade light. Gel blot analyses of FIN219, PHYA, and COP1 protein levels in wild-type Col **(A)**, *fin219* mutants **(B)**, and *pGR219* **(C)** in response to white and simulated shade light. Seedlings were germinated and grown under white light (high R:FR) for 2 days, then kept in white light or transferred to simulated shade (low R:FR) for 4 days. Total protein of 150 μg was loaded in each lane and probed with FIN219, PHYA and α-tubulin monoclonal and COP1 polyclonal antibodies. α-tubulin (TUB) was a loading control. Data are mean ± SE from two independent experiments. The asterisk indicates a non-specific band. Different lowercase letters represent significant differences by ANOVA at *P* < 0.05.

The onset of shade avoidance is coupled with transcriptional and translational reprogramming. FIN219 (AtGH11.3) is a GH3 family protein, which is rapidly upregulated in response to auxin (Hsieh et al., 2000). Recently, *GH3.3* was found induced by simulated shade (Crocco et al., 2015). We used quantitative real-time PCR (qRT-PCR) to determine the effect of simulated shade on *FIN219* transcript levels. Simulated shade did not alter *FIN219* expression in the wild type and *fin219* mutants; however, *FIN219* mRNA level was induced in *pGR219* (Supplementary Figure S1). Furthermore, we used protein gel blot analysis to demonstrate how shade affects FIN219 protein levels. FIN219 protein level was significantly reduced under simulated shade as compared with white light both in the wild type and *pGR219* (**Figures 2A–C**). Under white light, FIN219 protein level was lower in the *fin219* mutants (the null mutant *fin219-2* and *jar1-1*) than in the wild type. Simulated shade further reduced FIN219 level in these mutants (**Figure 2B**). Thus, shade light may reduce FIN219 protein level, and overaccumulation of FIN219 protein in *pGR219* (**Figure 2C**) may inhibit shade responses (**Figures 1A–C**).

We further determined the effect of FIN219 on PHYA and COP1 protein levels under simulated shade. In agreement with previous reports (Martínez-García et al., 2014), under shade light, PHYA protein level was increased in the wild type and slightly reduced in *fin219* mutants (**Figures 2A,B**). However, the *pGR219* line showed spontaneous accumulation of high levels of PHYA under white light, with no enhanced accumulation under simulated shade (**Figure 2C**). Thus, *pGR219* line may be insensitive to shade light.

CONSTITUTIVE PHOTOMORPHOGENIC 1 is essential for hypocotyl and petiole elongation under shade light (Rolauffs et al., 2012). COP1 level does not change with exposure to shade, and COP1 nuclear accumulation is required for full shade responses (Pacín et al., 2013). To determine how FIN219 affects COP1 level, we measured COP1 protein levels. As expected, COP1 level did not change largely under shade light; however, under white light and shade, COP1 level was greater in *fin219* mutants and *pGR219* than the wild type (**Figures 2B,C**). FIN219-altered COP1 may in turn affect the shade responses.

### *fin219-2*-Mediated Shade Responses Are Independent of phyA-Mediated High-Irradiance Response

**FIGURE 3:**
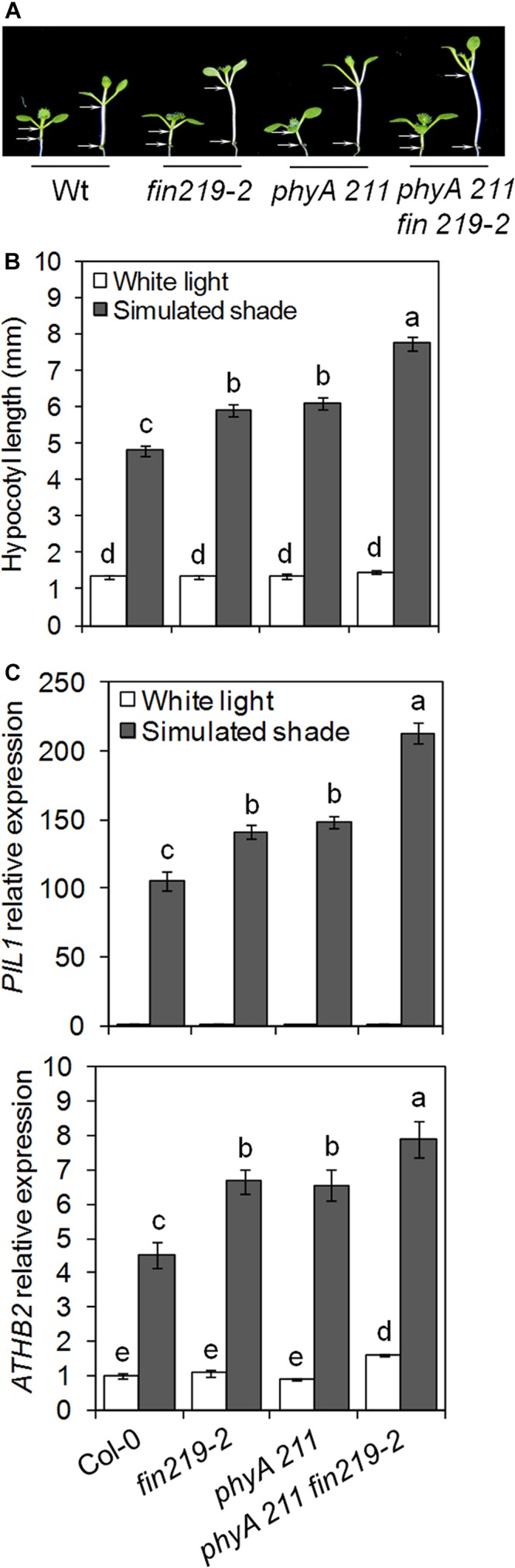
FIN219 and phyA have a synergistic effect on shade-mediated hypocotyl elongation and gene expression. **(A)** Seedlings of *fin219-2, phyA 211* and the double mutant *phyA 211 fin219-2* were grown under white light (high R:FR) or simulated shade (low R:FR) for 4 days. **(B)** Quantification of hypocotyl lengths of seedlings shown in **A**. Data are mean ± SE (*n* = 25). **(C)** qRT-PCR of *PIL1* and *ATHB2* expression in seedlings grown under white light or simulated shade for 4 days standardized to the wild type under white light. Data are mean ± SE from three biological replicates. Different lowercase letters represent significant differences by ANOVA at *P* < 0.05.

The *phyA* mutant exhibits a long hypocotyl phenotype under continuous FR light or continuous white light supplemented with FR light. This response of *phyA* is due to the FR high-irradiance response (HIR) (Johnson et al., 1994; Yanovsky et al., 1995; Martínez-García et al., 2014). FIN219 is a component of phyA-mediated FR light signaling (Hsieh et al., 2000). FIN219-mediated shade responses may be due to phyA-mediated HIR signaling. To clarify this possibility, we analyzed phenotypic responses of the *phyA211 fin219-2* to shade (Supplementary Figures S2A,B). Shade-induced hypocotyl elongation and gene expression were measured in *fin219-2* and *phyA 211* single mutants and *phyA211 fin219-2* double mutants under white light and simulated shade. Under simulated shade, hypocotyl elongation and the expression of shade-induced marker genes such as *PIL1* and *ATHB2* was greater in the double mutant than each of the single mutants (**Figures 3A–C**), which suggests that FIN219-mediated shade response is independent of phyA-mediated FR-HIR.

**FIGURE 4:**
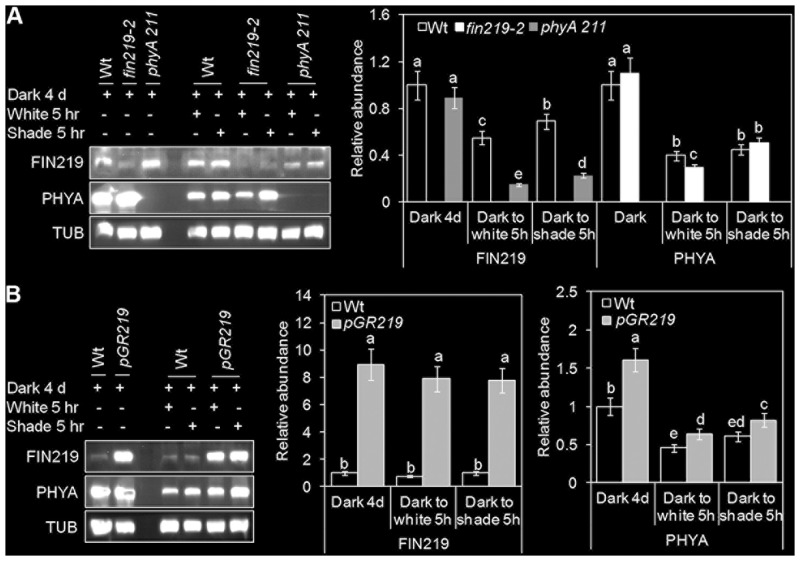
FIN219 and phyA regulate each other in response to shade. Gel blot analyses of FIN219 and PHYA transient levels in transition from dark to shade. Seedlings of wild-type (Wt), *fin219-2* and *phyA 211* **(A)** or Wt and *pGR219* **(B)** were grown in the dark for 4 days, transferred to white light or simulated shade for 5 h, and then subjected to protein gel blot analyses. Data are mean ± SE protein abundance from two independent experiments. Different lowercase letters represent significant differences by ANOVA at *P* < 0.05.

We showed reduced FIN219 level under shade light. However, in a dark-to-light transition experiment, FIN219 level in the wild type was reduced under light as compared with under dark, with a significant increase in FIN219 level under white and shade light (**Figure 4A**). Intriguingly, FIN219 level was lower in *phyA* mutants than the wild type under all light conditions (**Figure 4A** and Supplementary Figure 2B) except dark (**Figure 4A**), which implies that phyA positively regulates FIN219 under short-term shade. Similarly, in contrast to light-grown seedlings (**Figures 2B,C**), in dark-grown seedlings transferred to white light for 5 h, PHYA level was lower in the *fin219*-2 mutant than the wild type, and under shade light, it was comparable in *fin219-2* and wild type (**Figure 4A**). However, under all light conditions examined, PHYA protein level was greater in *pGR219* than the wild type (**Figure 4B**). FIN219 and phyA may regulate each other depending on the genotype and light condition, and output of this regulation may depend on a specific light condition.

### *FIN219* Differentially Alters the Expression of *PIF5* and *PAR1*, for Altered Auxin Responses

**FIGURE 5:**
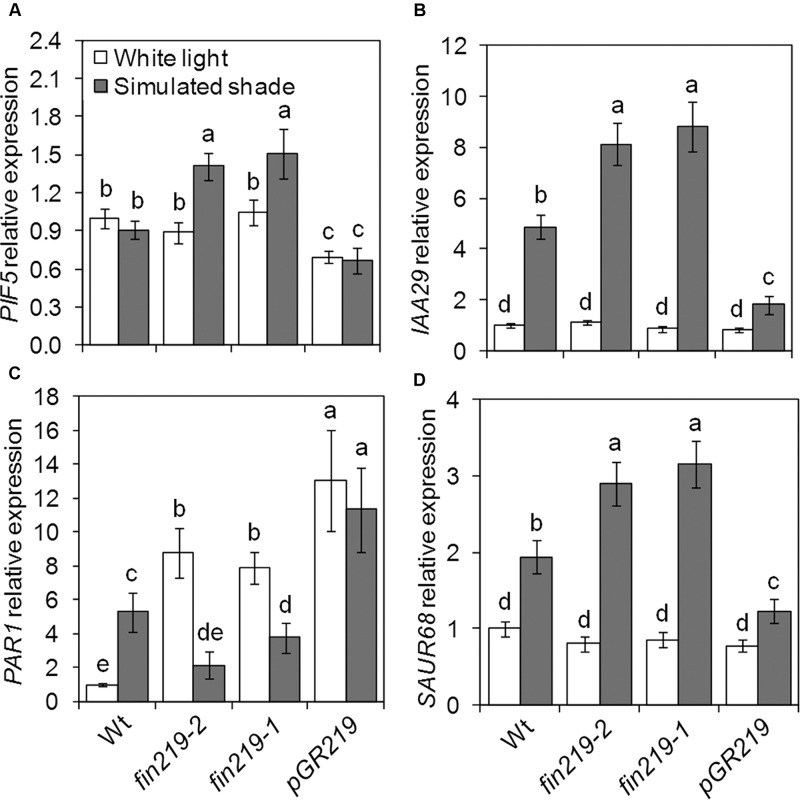
*FIN219* differentially regulates transcription factors *PIF5* and *PAR1* under shade. qRT-PCR analysis of *PIF5* **(A)**, *IAA29* **(B)**, *PAR1* **(C)**, and *SAUR68* **(D)** expression in seedlings grown under white light or simulated shade for 4 days standardized to the wild type under white light. Data are mean ± SE from three biological replicates. Different lowercase letters represent significant differences by ANOVA at *P* < 0.05.

**FIGURE 6:**
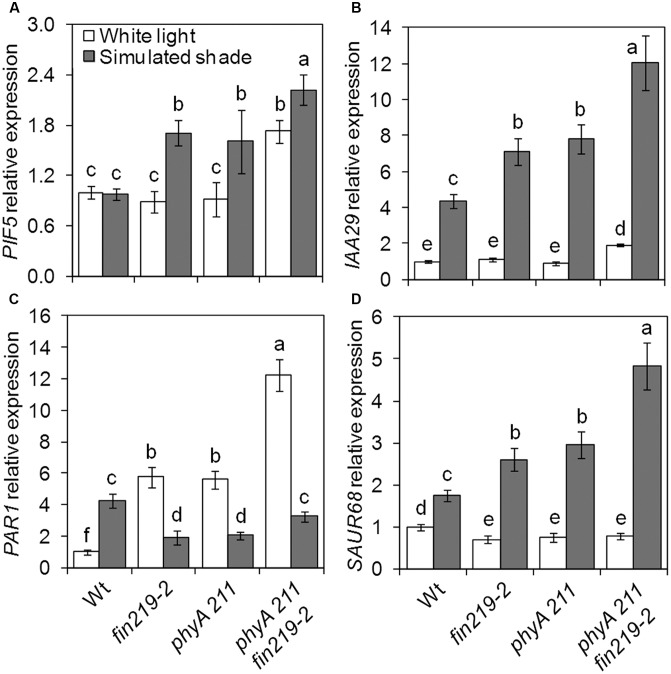
*FIN219* and phyA regulates key shade regulators. qRT-PCR analysis of *PIF5* **(A)**, *IAA29* **(B)**, *PAR1* **(C)**, and *SAUR68* **(D)** expression in seedlings of wild type (Wt), *fin219-2*, *phyA 211*, and *fin219-2phyA 211* grown under white light or simulated shade for 4 days standardized to the wild type under white light. Data are mean ± SE from three biological replicates. Different lowercase letters represent significant differences by ANOVA at *P* < 0.05.

Several groups of TFs are involved in shade light signaling. In our recent study, we found that *FIN219* affects a number of bHLH TFs (Chen et al., 2015). While checking the expression of shade signaling genes, we found that the bHLH TFs *PIF5* and *PAR1* were affected by *FIN219*. As expected, *PIF5* expression in the wild type did not differ under simulated shade and white light (**Figure 5A**). Disruption of *FIN219* expression by mutation (*fin219-2* and *fin219-1*) significantly induced *PIF5* expression under simulated shade, which was decreased in *pGR219* regardless of light condition (**Figure 5A**). Similarly, the effect of *FIN219* on *PAR1* expression was significantly pronounced under both white and shade light. Under white light, *PAR1* expression was greater in *fin219* mutants and *pGR219*, whereas under shade light, *PAR1* expression was reduced in *fin219* mutants and remained unchanged in *pGR219* (**Figure 5C**). Both *PIF5* and *PAR1* affect auxin biosynthesis and signaling under shade light (Roig-Villanova et al., 2007; Hornitschek et al., 2012). To check how their altered expression was translated into downstream effects, we evaluate the expression patterns of auxin-response genes *IAA29* and *PAR1* target *SAUR68.* The expression of both *IAA29* and *SAUR68* was enhanced in *fin219* mutants and reduced in *pGR219* under shade (**Figures 5B,D**). In addition, *PIF5* showed high induction and *PAR1* reduced expression in the *phyA 211 fin219-2* double mutant under shade (**Figures 6A,C**). Moreover, the expression of both *IAA29* and *SAUR68* was synergistically increased in *phyA 211fin219-2* under simulated shade (**Figures 6B,D**). Thus, FIN219 modulates auxin homeostasis under simulated shade through *PIF5* and *PAR1*.

### FIN219 Reduces Nuclear Accumulation of COP1 through Direct Physical Interaction under Shade

The *cop1* mutants show very short hypocotyls under high and low R:FR conditions. The *cop1-4* mutant still shows a residual shade response (Pacín et al., 2013), which is not observed in *cop1-6* (Supplementary Figures S3A,B). To demonstrate how *fin219* mutation affects *cop1* mutants under simulated shade, we assessed the double mutants *cop1-4 fin219-2* and *cop1-6 fin219-2* in response to simulated shade. Shade-induced hypocotyl elongation was significantly enhanced although slightly in *cop1-4 fin219-2* as compared with *cop1-4*, with no difference between *cop1-6 fin219-2* and *cop1-6* (Supplementary Figures S3A,B).

**FIGURE 7:**
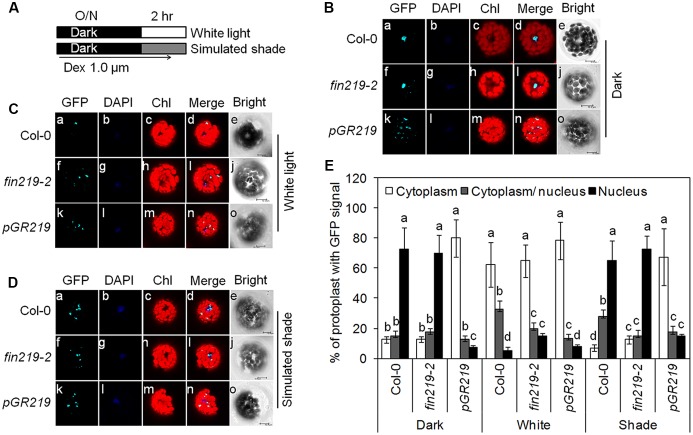
FIN219 overexpression is able to exclude GFP-COP1 from the nucleus to the cytoplasm in response to shade. **(A)** A scheme to illustrate experimental conditions of the protoplasts. Transformed *Arabidopsis* mesophyll protoplasts by *35S:GFP-COP1* were incubated at 22°C overnight in the dark **(B)** and the next day transferred to white light **(C)** or simulated shade **(D)** for 1 h. Bars = 10 μm. **(E)** Quantification of the percentage of protoplasts with GFP signals in different cellular compartments. Data are mean ± SE from two biological replicates. Different lowercase letters represent significant differences by ANOVA at *P* < 0.05.

Enhanced COP1 nuclear accumulation is essential for shade responses (Pacín et al., 2013). Previous studies showed that FIN219 interacted with COP1 under dark and FR light. Moreover, *FIN219* overexpression can exclude COP1 from the nucleus to the cytoplasm even in the dark (Wang et al., 2011). To further investigate the effect of FIN219 on COP1 nuclear localization under white and shade light, we performed protoplast transient assays using *Arabidopsis* mesophyll protoplasts from the wild type, *fin219-2* and *pGR219* and the construct *p35S: GFP-COP1* in a binary vector. Under the dark, *GFP-COP1* localized in the nucleus in wild-type protoplasts and *fin219-2* (**Figures 7Ba–i,E**) but mostly in the cytoplasm in *pGR219* protoplasts (**Figures 7Bk–n,E**). Under white light, *GFP-COP1* signal is mostly abundant in the cytoplasm in wild type, *fin219-2* and *pGR219* protoplasts (**Figures 7Ca–d,f–i,k–n,E**). Upon exposure to simulated shade for 1 h, *GFP-COP1* signals mainly concentrated in the nucleus in wild type and *fin219-2* (**Figures 7Da–d,f–i,E**). However, overexpression of FIN219 in *pGR219* inhibited COP1 nuclear accumulation (**Figures 7Dk–n,E**). Therefore, FIN219 overexpression can induce COP1 accumulation in the cytoplasm in response to simulated shade.

**FIGURE 8:**
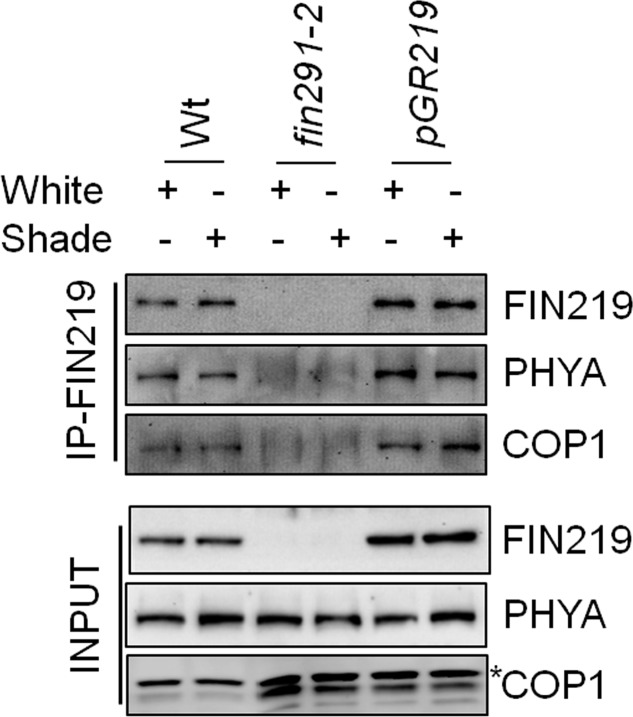
FIN219 physically interacts with phyA and COP1 under shade light. Co-immunoprecipitation analysis of FIN219 interacting with phyA and COP1 in indicated genotypes under shade light. In total, 2 mg protein extracts from Col-0 (Wt), *fin219-2* and *pGR219* seedlings grown under white and shade light for 2 days were mixed with FIN219 monoclonal antibodies and then immunoprecipitated. The pellets were used for SDS-PAGE and protein gel blot analysis. The probes are FIN219 and PHYA monoclonal antibodies and COP1 polyclonal antibody. The asterisk indicates a non-specific band.

Furthermore, to determine the possibility of physical interaction between FIN219 and COP1 under shade, we used co-immunoprecipitation with wild type, *fin219-2* and *pGR219* seedlings grown under simulated shade. FIN219 directly interacted with COP1 and phyA (**Figure 8**). Interaction of both molecules was stronger in the *FIN219* overexpression line *pGR219*, which suggests that FIN219 abundance may affect the degree of interactions (**Figure 8**). Hence, FIN219 can regulate the subcellular localization of COP1 under white and simulated shade, and lack of FIN219 regulation of COP1 in *fin219* mutants compared to the wild type makes it more sensitive to shade light.

## Discussion

Hypocotyl elongation is a key adaptation of *Arabidopsis* seedlings to avoid shade light. Here, we report that FIN219/JAR1, a JA-conjugating enzyme, plays a negative role in regulating shade responses such as hypocotyl elongation and expression of shade components. The double mutant *phyA 211fin219-2* had a synergistic effect in response to simulated shade, which suggests that phyA and FIN219/JAR1 work in parallel pathways to regulate shade signaling, which is consistent with the additive levels of TFs *PIL1* and *ATHB2* and auxin-response genes *IAA29* and *SAUR68* under the same condition. Moreover, FIN219/JAR1 and PHYA levels were down- and upregulated by shade, respectively. Intriguingly, PHYA and COP1 levels were affected in the *fin219* mutants under simulated shade. In particular, FIN219/JAR1 overexpression under shade could change COP1 subcellular localization from the nucleus to the cytoplasm. Further Co-IP studies under shade revealed that FIN219/JAR1, phyA and COP1 interacted with each other. These data indicate that FIN219/JAR1 plays a vital role in regulating shade responses likely by modulating the expression and subcellular location of shade components.

FIN219/JAR1 is a JA-conjugating enzyme, responsible for the formation of JA-Ile and involved in the regulation of plant development and defense responses likely via crosstalk with different hormones and TF-mediated signaling pathways (Chen et al., 2015). Mutation in the *FIN219/JAR1* locus resulted in enhanced shade-induced hypocotyl elongation with different degrees (**Figures 1A,B**) (Robson et al., 2010), whereas its overexpression led reduced elongation responses (**Figures 1A,B**), which suggests a negative role of *FIN219* in shade signaling. Since *FIN219* is an auxin and JA inducible gene and its function in JA signaling is well known, how it modulates other hormone signaling pathways is largely unknown. Our previous studies also indicated that FIN219 could crosstalk with other different hormones (Chen et al., 2015). The *fin219-2* is a T-DNA insertion knockout mutant (Wang et al., 2011), whereas *jar1-1* is an EMS mutant (Staswick et al., 2002) and *fin219-1* is a mis-regulated mutant with changes of methylation status in the promoter of its gene (Hsieh et al., 2000). Thus, *jar1-1* and *fin219-1* mutants contain reduced levels of proteins compared to wild type. It could be possible that in these mutant lines (*jar1-1* and *fin219-1*), FIN219 perturbs other hormone signaling pathways and makes plants more sensitive to shade. This is our hypothesis that needs further investigation to clarify. In addition, COP1 is necessary for hypocotyl and petiole elongation under shade light (Rolauffs et al., 2012). Furthermore, B-box-containing 21 (BBX21) acts downstream of COP1 to negatively regulate the shade response (Crocco et al., 2010). FIN219/JAR1 appears to affect COP1 levels under shade (**Figures 2B,C**). Intriguingly, *BBX21* negatively regulated *FIN219* transcript levels under canopy shade (Crocco et al., 2010), so FIN219-COP1-BBX21 may form a regulatory feedback loop in response to shade environments. Recent studies revealed that phyB and PIFs form a mutually negative feedback loop under continuous red but not shade light (Leivar et al., 2012a). Thus, a local loop regulation initiated by FIN219-COP1-BBX21, leading to reduced levels of active JA-Ile, may play a vital role in fine-tuning shade light signaling.

FIN219/JAR1 acts as a major enzyme for the formation of physiologically active JA-Ile to regulate plant growth and defense responses (Staswick et al., 2002; Wang et al., 2011). Its protein levels should be strictly modulated in response to developmental and environmental cues. So far, multiple photoreceptors, including phyA, and different hormones such as auxin and JAs can regulate FIN219 levels. Current work indicated that *pGR219* with *FIN219* overexpression has always severely stunted phenotype (**Figure 1**). The expression levels of *IAA29* and *SAUR68* in *pGR219* are always similar with Col-0 under white light (**Figures 5B,D**). Moreover, PIF5 and PAR1 have opposing effects on shade responses, which suggests that FIN219 may trigger major effects on shade-responsive genes mainly under shade conditions. Besides, PIF5 positively regulates and PAR1 negatively affects shade responses. In *pGR219*, the *PIF5* expression is less and *PAR1* expression is greater than wild type (**Figures 5A,C**). However, these gene expressions do not alter with response to shade in *pGR219*. In addition, our previous studies indicated that *FIN219* overexpression could exclude COP1 from the nucleus to the cytoplasm even in the dark without greatly altering COP1 level (Wang et al., 2011). Shade also resulted in COP1 accumulation in the nucleus (**Figures 7D,E**). In contrast, *pGR219* under shade showed more COP1 accumulation in the cytoplasm and substantial reduction of COP1 in the nucleus compared to wild-type Col-0 under the same condition, which suggests that the accuracy of COP1 subcellular location is critical for shade responses in addition to expression of shade components. Thus, the *PGR219* with pronounced short-hypocotyl phenotype under white light and shade conditions may have substantial effects on plant growth and development in addition to light signaling.

Shade light illumination affects a number of genes, including TFs (Sessa et al., 2005; Roig-Villanova et al., 2006). PIF-related TFs are essential for shade-induced responses (Lorrain et al., 2008; Casal, 2013). As expected, wild-type *PIF5* transcript levels did not change greatly under shade light. However, *FIN219* negatively regulated *PIL1* and *PIF5* gene expression under low R:FR light (**Figures 1C**, **5A**). As well, the expression of several TFs such as *HFR1, ATHB2*, *PIL1*, and *PAR1/2* were rapidly induced under shade light (Carabelli et al., 1996; Salter et al., 2003; Sessa et al., 2005; Roig-Villanova et al., 2006). HFR1 and PAR1/2 negatively regulate shade responses (Roig-Villanova et al., 2007; Hornitschek et al., 2009), whereas ATHB2 and PIL1 are positive (Steindler et al., 1999; Salter et al., 2003) and negative regulators, respectively (Li et al., 2014). *PAR1* expression was positively regulated by *FIN219* under shade (**Figure 5C**). Therefore, FIN219 may regulate the expression of early shade-induced genes positively and negatively. In addition, *PIF5* and *PAR1* modulate auxin biosynthesis and signaling (Roig-Villanova et al., 2007; Hornitschek et al., 2012). In agreement, the expression of auxin-response genes *IAA29* and *PAR1* target *SAUR68* is associated with *PIF5* and *PAR1* expression, respectively. As well, the *PIF5* and *PAR1* expression was higher in the double mutant *phyA211 fin219-2* than in each single mutant under shade (**Figures 6A,C**), so FIN219 may act independently of phyA-mediated signaling to modulate auxin signaling in response to shade.

In addition, *PAR1* transcripts are repressed by *PHYA* and *PHYB* overexpression under simulated shade (Roig-Villanova et al., 2006). Its transcripts are also suppressed by phyA and phyB under FR and R light, respectively. The PHYA protein level in *fin219* mutants was similar to that in wild type (**Figure 2B**) and it was greater in *PGR219* under white light (**Figure 2C**), which suggests that *PAR1* transcripts would be less than wild type under white light. However, in **Figure 5C**, *PAR1* expression was even greater in both *fin219* mutants and *PGR219* than in wild type under white light, which implies that *PAR1* expression patterns in *fin219* mutants and *PGR219* may involve hormone effects such as JAs and auxin. The detailed mechanisms remain elusive.

Phytochromes were shown to play vital roles in shade signaling (Devlin et al., 1999; Franklin and Whitelam, 2005; Casal, 2012). phyB is stable in light and functions as a major photoreceptor in suppression of shade-mediated hypocotyl elongation. Under a high R:FR ratio, the phenotype of the *phyB* mutant was similar to the wild type under shade. Furthermore, phyD and phyE were redundant to phyB in repressing shade responses, whereas phyC did not play a role in shade responses (Franklin and Quail, 2010). Functional roles of phyA involved in the repression of shade responses remain largely unknown. Here, we found that PHYA levels were increased by simulated shade (low R:FR ∼ 0.05) (**Figures 2**, **4**), which agrees with the report by Martínez-García et al. (2014). An increase in PHYA level on exposure to shade even for 5 h (**Figure 4**) is likely related to the phyA function in fine-tuning the chlorophyll biosynthesis in response to partial shading (Brouwer et al., 2014). Moreover, phyA appears to positively regulate FIN219 levels in short-term exposure to shade (**Figure 4A**), rather than long-term exposure to shade (Supplementary Figure S2B). In turn, increased FIN219 levels leading to enhanced JA-Ile levels resulted in reduced the chlorophyll content under shade. This speculation is consistent with JAs being able to decrease chlorophyll content and reduce photosynthesis in light (He et al., 2002; Zhai et al., 2007). In contrast, with longer exposure to shade, FIN219 level was reduced in the wild type (**Figure 2** and Supplementary Figure S2B) and positively regulated PHYA level (**Figure 2B**). Thus, reduced FIN219 level under shade may involve other mechanisms, and both FIN219 and phyA may regulate each other in response to low R:FR light.

CONSTITUTIVE PHOTOMORPHOGENIC 1 is a negative regulator of photomorphogenesis and encodes an E3 ubiquitin ligase. Under the dark, COP1 accumulates in the nucleus and results in the degradation of positive regulators such as HY5 of photomorphogenesis. Upon light exposure, COP1 is inactivated by migrating to the cytoplasm and other unknown mechanisms. Shade light can trigger COP1 accumulation rapidly in the nucleus, and it positively modulates the shade responses (Pacín et al., 2013). Moreover, COP1 physically interacts with FIN219 under the dark and continuous FR light and restricts its nuclear accumulation on overexpression of FIN219 (Wang et al., 2011). We found COP1 mainly accumulated in the cytoplasm and unable to migrate to the nucleus in *pGR219* under the conditions examined, including shade for 1 h (**Figures 7B–E**), which significantly affects hypocotyl elongation (**Figures 1A,B**). Thus, abundant FIN219 protein affects the total content of active COP1 protein in the nucleus. Ethylene promotes hypocotyl elongation in the light by triggering COP1 nuclear accumulation, which enhances HY5 degradation (Yu et al., 2013). FIN219 levels responsible for the formation of JA-Ile, an active form of JA, are likely tightly regulated in response to low R:FR to modulate the subcellular localization of COP1.

FIN219/JAR1 is a cytoplasmic protein in FR light and remains in the same location even under shade (Hsieh et al., 2000; **Figure 7**). Co-IP studies indicated that FIN219/JAR1 dose-dependently interacted with phyA and COP1 proteins under shade (**Figure 8**). *FIN219* overexpression excluded COP1 from the nucleus to the cytoplasm under FR light (Wang et al., 2011). As well, ectopic expression of FIN219 under low R:FR light changed the COP1 subcellular location from the nucleus to the cytoplasm because COP1 was localized in the nucleus under shade light. However, FIN219 level in the wild type was reduced by shade light (**Figure 2**) and was increased by 5-h shade as compared with white light (**Figure 4**). FIN219 and COP1 interaction likely occurred in the cytoplasm at the early stage of shade light exposure. This speculation is consistent with transient expression at 1-h shade that resulted in more accumulation of GFP-COP1 in the cytoplasm by ectopic expression of FIN219 (*pGR219*) (**Figure 7**).

In addition, the levels of PHYA were increased and stabilized by continuous and transient shade light (**Figures 2**, **4**), which leads to suppression of hypocotyl elongation, with an antagonistic effect on phyB deactivation by low R:FR light. PhyA and phyB likely function dynamically to modulate hypocotyl elongation in response to changes in R:FR ratios in natural environments. In addition, FIN219 and phyA positively regulated each other under shade (**Figures 2**, **4**, **8**). phyA is activated by FR light and migrates to the nucleus. Whether FIN219 affects phyA subcellular localization, leading to their interaction in the cytoplasm upon exposure to shade light, remains to be elucidated.

**FIGURE 9:**
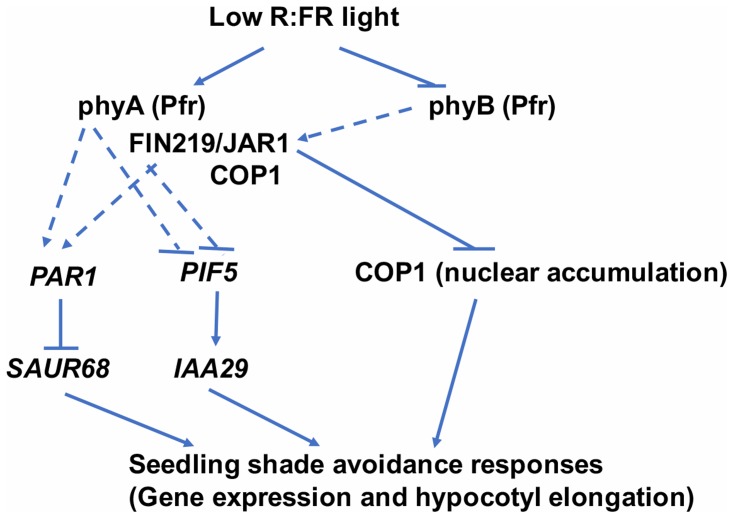
A model to illustrate FIN219 functions in regulating shade responses. Low R:FR light perceived by the photoreceptor phyB regulates specific gene expression likely via FIN219 associated complex (phyA-FIN219/JAR1-COP1)-mediated positive or negative modulation of transcription factors, including *PAR1* and *PIF5*. As well, FIN219 regulated by phyA through direct interaction interacts with COP1 and inhibits its nuclear accumulation. All these events lead to shade-induced gene expression and hypocotyl elongation. Regular arrow, positive effect; inverted T, negative effect. Dashed lines represent an indirect regulation.

FIN219/JAR1 is a JA-conjugating enzyme and interacts with multiple partners, including COP1 (Chen et al., 2007; Wang et al., 2011; **Figure 8**). These data suggest that FIN219 may have a dual function with an enzymatic and protein–protein interaction activities. Collectively, our data show that FIN219/JAR1, a JA-conjugating enzyme, functions as a negative regulator in shade signaling and may work with phyA and COP1 in response to shade. Alternatively, low R:FR ratio may inactivate phyB by reducing its abundance in the nucleus and decrease FIN219/JAR1 levels, thereby leading to increased PIF5 level and reduced PAR1 level and increased COP1 accumulation in the nucleus. These events activate downstream genes such as *IAA29* and *SAUR68* and shade responses, including hypocotyl elongation (**Figure 9**).

## Author Contributions

SS and H-LH designed the experiments; SS and H-WJ performed experiments; SS and H-LH did data analyses; SS and H-LH wrote the manuscript.

## Conflict of Interest Statement

The authors declare that the research was conducted in the absence of any commercial or financial relationships that could be construed as a potential conflict of interest.

## Footnotes

1 http://rsb.info.nih.gov/
