# Supplementary material for: FAR-RED INSENSITIVE 219/JAR1 Contributes to Shade Avoidance Responses of Arabidopsis Seedlings by Modulating Key Shade Signaling Components
Source: Front Plant Sci. 2017 Nov 2;8:1901. doi: 10.3389/fpls.2017.01901 (PMC5673645; doi:10.3389/fpls.2017.01901)
Supplement: Supplementary file 1 [file Image_1.PDF]

SUPPLEMENTARY MATERIAL

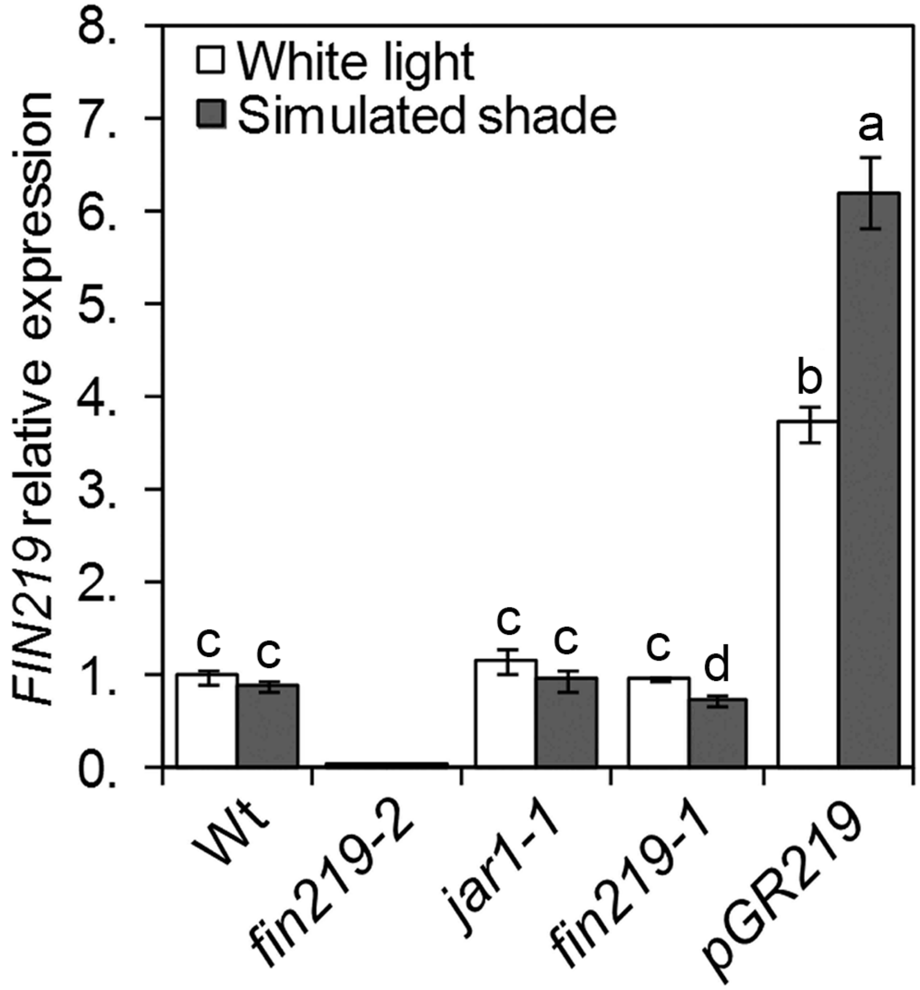

Figure 1 | *FIN219* expression in wild type, *fin219* mutants and overexpression lines under shade light. qRT-PCR analysis of *FIN219* expression in seedlings of wild type (wt), *fin219* mutants and overexpression lines grown under white light or simulated shade for 4 days standardized to the wild type under white light. Data are mean±SE from 3 biological replicates. Different lowercase letters represent significant differences by ANOVA at $P < 0.05$.

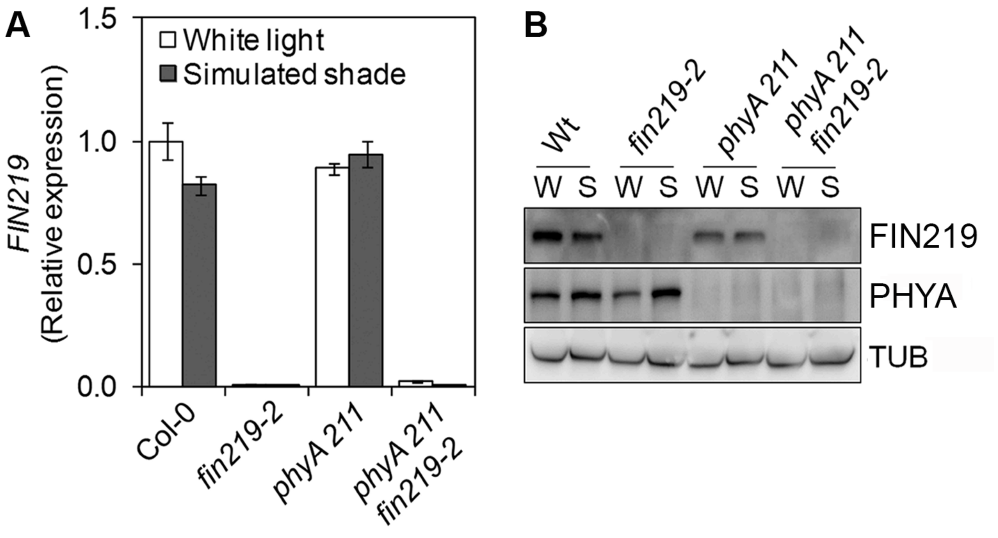

Figure 2 | FIN219 and phyA regulates each other under shade light. (A) qRT-PCR analysis of *FIN219* expression in seedlings of wild-type Col-0, *fin219-2*, *phyA 211*, and *phyA 211 fin219-2* mutants grown under white light or simulated shade for 4 days standardized to the wild type under white light. Data are mean±SE from 3 biological replicates. **(B)** Gel blot analysis of FIN219 and PHYA protein levels in wild type, *fin219-2, phyA 211 and phyA 211 fin219-2* in response to white and simulated shade light.

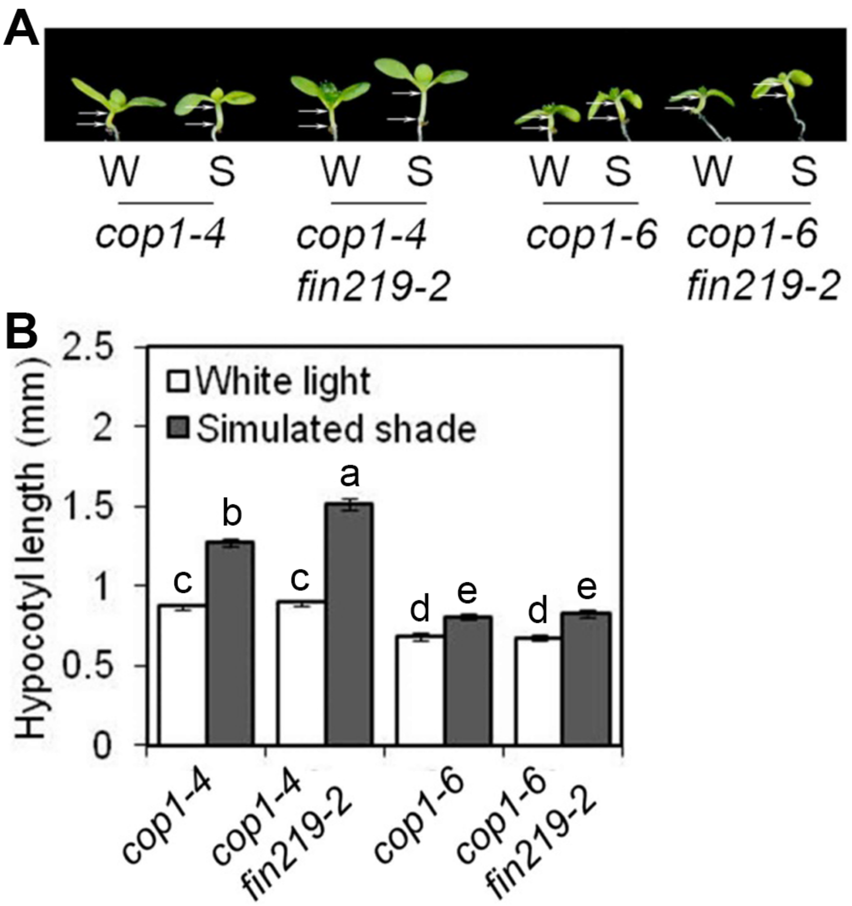

Figure 3 | *fin219-2* enhanced *cop1-4* response to simulated shade. (A) Phenotypic responses of *cop1* mutants (*cop1-4* and *cop1-6*) and *cop1 fin219-2* double mutant seedlings grown under white light (high R:FR) or simulated shade (low R:FR) for 4 days. **(B)** Quantification of hypocotyl lengths of seedlings shown in **(A)**. Data are mean±SE (n= 25). Different lowercase letters represent significant differences by ANOVA at $P < 0.05$.

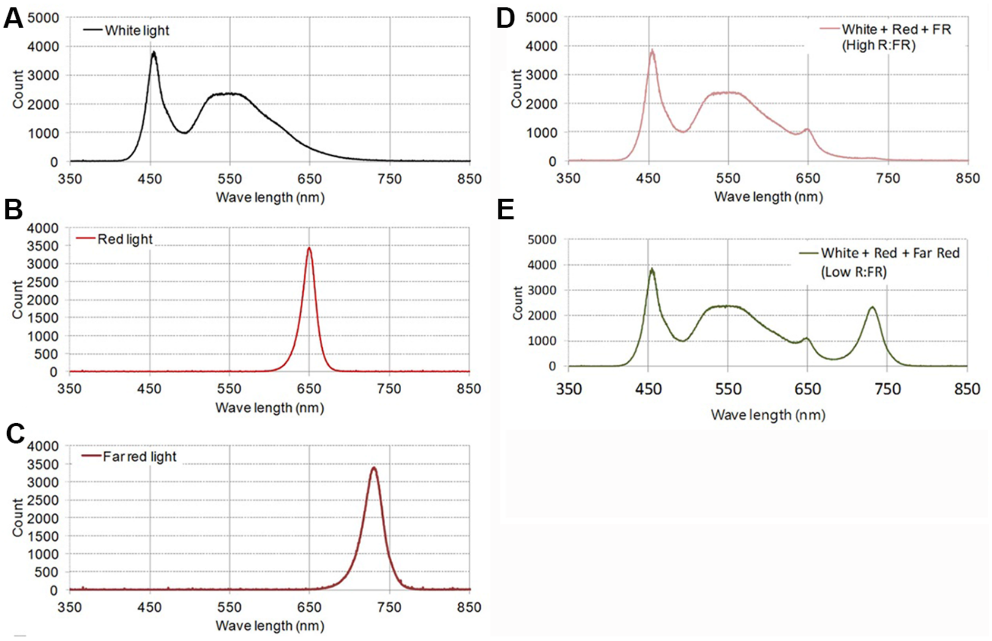

Figure 4 | Light spectrum of LED light used in this study. LED white light does not contain red and FR light wavelengths. High R:FR light is the combination of white, red (5.21 µmol $m^{-2}s^{-1}$) and FR light (2.25 µmol $m^{-2}s^{-1}$) LED. Low R:FR light is the combination of white, red (R5.21 µmol $m^{-2}s^{-1}$) and FR light (80.02 µmol $m^{-2}s^{-1}$) LED.

Table S1. Primer pairs used for quantitative real-time PCR.

Gene	Accession number	primer sequences (5′ →3′)
ACT2	AT3G18780	F -TGAAGTGTGATGTGGATATCAGG<br>R -GTGATTTCTTTGCTCATACGGTC
FIN219	AT2G46370	F - CAACTGTTTAGACCGAGCATTCA<br>R - CAACTGTTTAGACCGAGCATTCA
PIL1	AT2G46970	F - GCAGCAACACCAACATCAATAC<br>R - GGAATTAGTCCACTTGGGTGATTA
ATHB2	AT4G16780	F - TGAGCCCACCCACTACTTTGAC<br>R - AGGAGCCCACGCATTGACC
PAR1	AT2G40200	F - TCATGCTCAGCCACCGTGAAATC<br>R - CCTTGACCTCATCTTCTTCTTCTTC
PIF5	AT2G42870	F - CACACTGACGAATCTGTATCTCTATC<br>R - ATCCTATCTCTCCTCCTCCTTTC
IAA29	AT4G32280	F - CTTCCAAGGGAAAGAGGGTGA<br>R - TTCCGCAAAGATCTTCCATGTAAC
SAUR68	AT1G29490	F - GGTTTACACTGCGGATCAAGTA<br>R - CCTTCTGTGGGAATACCAAACT
